# Conservative Management of Placenta Percreta With Perforation in an Infertile Lupus Patient Following Embryo Transfer: A Report of a Rare Case

**DOI:** 10.7759/cureus.74774

**Published:** 2024-11-29

**Authors:** Mika Sugihara, Kuniaki Ota, Toshifumi Takahashi, Keitaro Tasaka, Hana Okamoto, Yumiko Morimoto, Shogo Kawamura, Wataru Saito, Hiroaki Tsubouchi, Yoshiaki Ota, Koichiro Shimoya

**Affiliations:** 1 Obstetrics and Gynecology, Kawasaki Medical School, Kurashiki, JPN; 2 Fukushima Medical Center for Children and Women, Fukushima Medical University, Fukushima, JPN

**Keywords:** assisted reproductive technology (art), glucocorticoid, hormone replacement treatment, placenta accreta spectrum (pas), placenta percreta

## Abstract

Placenta accreta spectrum (PAS) is a life-threatening condition characterized by abnormal placental invasion of the myometrium and is often associated with uterine surgery. However, it can also occur in unscarred uteri, particularly during pregnancies using assisted reproductive technology (ART). Following a successful pregnancy via vitrified-warmed embryo transfer, a 33-year-old nulliparous woman with systemic lupus erythematosus and long-term steroid use presented with intra-abdominal hemorrhage due to placenta percreta and spontaneous uterine perforation at week 10 of gestation. The patient was managed conservatively to preserve fertility. Over two years, the placenta spontaneously resorbed, and laparoscopic repair of an anatomical defect in the muscular layer of the perforation was successfully performed. Finally, the uterus returned to normal size. This case underscores the increased risk of PAS in ART pregnancies, particularly during hormone replacement therapy cycles and long-term oral steroid administration, and highlights the potential for conservative management and fertility preservation following PAS with uterine perforation.

## Introduction

Placenta accreta spectrum (PAS) is a condition in which the placenta abnormally adheres to the myometrium, often due to a defect in the endometrial-myometrial interface [1]. This allows the chorionic villi and trophoblasts to invade the myometrium, leading to varying degrees of invasion, such as accreta, increta, or percreta, which can cause severe hemorrhage, morbidity, and even mortality.

This condition results from abnormal placental invasion through the entire uterine wall, weakening it and leading to rupture. Affected patients typically present with severe abdominal pain, vaginal bleeding, and potential signs of hypovolemic shock [2,3]. Diagnosis is challenging and often requires imaging or exploratory surgery. Management typically involves emergency laparotomy, hemorrhage control, and eventually hysterectomy may have to be performed to rescue the patient [3,4].

PAS is commonly seen in women with risk factors such as prior uterine surgery or cesarean section, but it can also occur in unscarred uteri. Recent studies have reported a higher incidence of placenta percreta following various assisted reproductive treatments [5,6].

Spontaneous uterine rupture with an unscarred uterus due to placenta percreta in the first trimester is rare and should be considered in the differential diagnosis of any pregnant woman presenting with acute abdominal pain and signs of shock even during early pregnancy [2,3]. In such cases, the prognosis is often serious, with significant risks of massive hemorrhage, shock, and maternal mortality, highlighting the critical importance of early diagnosis and timely surgical intervention [3,4].

Herein, we report a case of intra-abdominal hemorrhage due to placenta percreta at 10 weeks of gestation in an unscarred uterus after vitrified-warmed embryo transfer in a woman who had been taking glucocorticoids for 20 years for systemic lupus erythematosus (SLE). The patient underwent nonsurgical management to preserve the uterus. After two years, the placenta was naturally reabsorbed and the uterus returned to normal size.

## Case presentation

**Figure 1:**
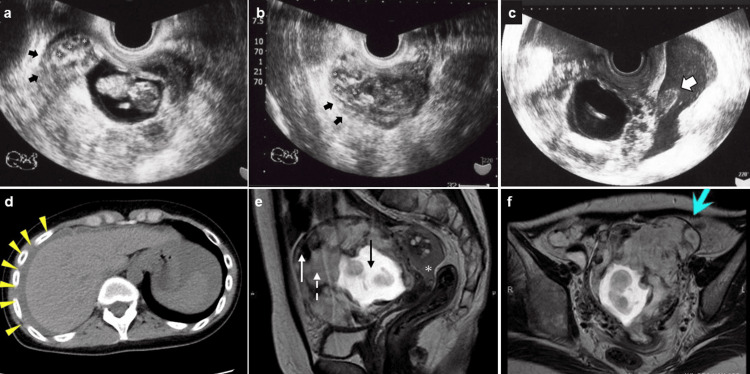
Sagittal sonographic image of the fundal placenta at 10 weeks of gestation Note the placental lacunae marked with asterisks (*) producing a “moth-eaten” appearance of the placenta (a) and the absence of the normal retroplacental sonolucency, or clearly detectable myometrium (black arrows) (b). Placental bulge protruding from the uterus is confirmed as bloody ascites (c). Bloody ascites around the liver, derived from unknown origin, are observed on abdominal computed tomography (yellow triangles) (d). The sagittal T2-weighted image shows the fetus (black arrow) and persistent high signal intensity of the placenta (dashed white arrow) with the low signal intensity of the adjacent myometrium (solid white arrow) with bloody ascites in the Douglas pouch (white asterisk) (e). Axial T2-weighted image shows a partial bulging of the placenta outside the uterus, a sign of “placental bulging” (blue arrow) (f).

The patient was a 33-year-old nulliparous Japanese woman with a history of SLE for 20 years, during which lupus enteritis developed. The patient had been taking oral prednisolone (7.5 mg/day) and tacrolimus (3 mg/day) since the initial diagnosis. The patient had no history of uterine surgery. After two years of unsuccessful attempts at natural conceiving, she underwent six cycles of timed intercourse with ovulation monitoring via transvaginal ultrasound. She then underwent in vitro fertilization because of unexplained infertility, resulting in 12 blastocysts being cryopreserved through vitrification. She underwent vitrified-warmed embryo transfer under hormone replacement treatment (HRT) on her third attempt and had a successful pregnancy. Owing to a pregnancy complicated by SLE, she was referred to our hospital for specialized perinatal care. Transvaginal ultrasonography revealed a singleton intrauterine pregnancy with a positive fetal heartbeat and crown-rump length of 23.5 mm, consistent with 10 weeks of gestation (Figure 1A). However, an abnormal placental structure with several small cystic areas was observed (Figure 1B). In contrast to trophoblastic disease, serum human chorionic gonadotropin (hCG) levels were 315,500 mIU/mL.

At 10 weeks and 6 days, she experienced a sudden onset of epigastric pain and hematemesis. Thus, she was emergently transferred to our hospital. Transvaginal ultrasound confirmed fetal heartbeat; however, small cystic areas around the placenta and ascites in the Douglas fossa were noted (Figure 1C). Her initial vital signs included a temperature of 38.5°C, heart rate of 110 bpm, blood pressure of 98/30 mmHg, and oxygen saturation of 99% on room air. However, her hemoglobin level on admission was 7.3 g/dL. She received emergency treatment, including eight units of packed red blood cells (RBCs) and eight units of fresh frozen plasma (FFP). Abdominal ultrasonography, computed tomography (CT), and upper gastrointestinal endoscopy were performed because of suspected gastrointestinal perforation caused by epigastric pain and hematemesis. However, abdominal CT revealed bloody ascites of unknown origin around the liver (Figure 1D). Because the cause of the bleeding remained unclear, her pain was manageable with analgesics, and her vital signs were stable, conservative treatment was pursued while investigating the bleeding source. On the third day of hospitalization, she received transfusions of eight units each of packed RBCs and FFP due to a drop in hemoglobin to 7.9 g/dL.

Her condition was unremarkable except for the presence of abnormal placental characteristics. On hospital day 18, fetal heart rate was absent, and repeat obstetric ultrasonography confirmed fetal death. On hospital day 20, contrast-enhanced magnetic resonance imaging (MRI) suggested placenta percreta, revealing placental villi invading the myometrium and a vascular mass protruding from the uterine serosa. Bleeding from the same site was suspected due to the presence of bloody ascites in the pelvis (Figure 1E, 1F). Based on these findings, a diagnosis of placenta percreta with bleeding from the perforated placental surface was made. We proposed several treatment options: hysterectomy, leaving the placenta in situ, removing as much of the placenta as possible, and surgically controlling the bleeding.

**Figure 2:**
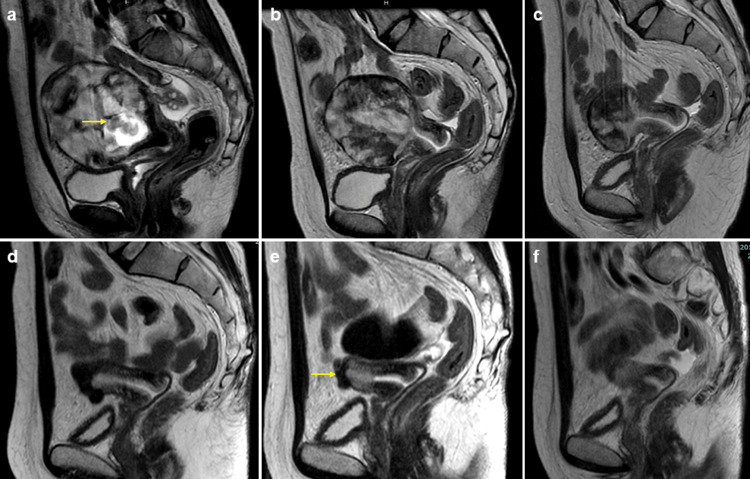
Sagittal T2-weighted contrast-enhanced magnetic resonance imaging (MRI) An indistinct border between the myometrium and placenta is shown, continuing to suggest placenta percreta, although the fetal (yellow arrow) and high-echoic placental components show signs of shrinkage (a). Further reduction in the fetal and placental components is confirmed, and the low-echoic normal myometrium is gradually becoming more defined (b). A follow-up MRI showed no abnormal blood flow in the area, and the low-echo normal myometrium became more distinct (c). The high echoic placental component has been completely replaced by low echoic myometrium, and the uterus has returned to its normal size (d). T2-weighted sagittal contrast-enhanced MRI shows an area of poor contrast at the uterine fundus and the thinning part of the myometrium, which is consistent with the site of the placental villi perforation into the myometrium (yellow arrow) (e). T2-weighted sagittal contrast-enhanced MRI shows that the myometrium has gradually recovered blood flow, and the muscle layer is maintained throughout the entire circumferential uterus (f).

She opted to wait for the natural delivery of the fetus and placenta to preserve fertility, with monitoring of serum hCG levels, despite being hospitalized owing to the high risk of sudden massive hemorrhage. On hospital day 36, she required an urgent transfusion of four units each of packed RBCs and FFP, along with uterine artery embolization (UAE) of the bilateral internal iliac and uterine arteries due to sudden massive genital bleeding. Contrast-enhanced MRI performed three days after UAE showed an indistinct border between the myometrium and placenta, suggesting placenta percreta, although the fetal and placental components showed signs of shrinkage (Figure 2A).

**Figure 3:**
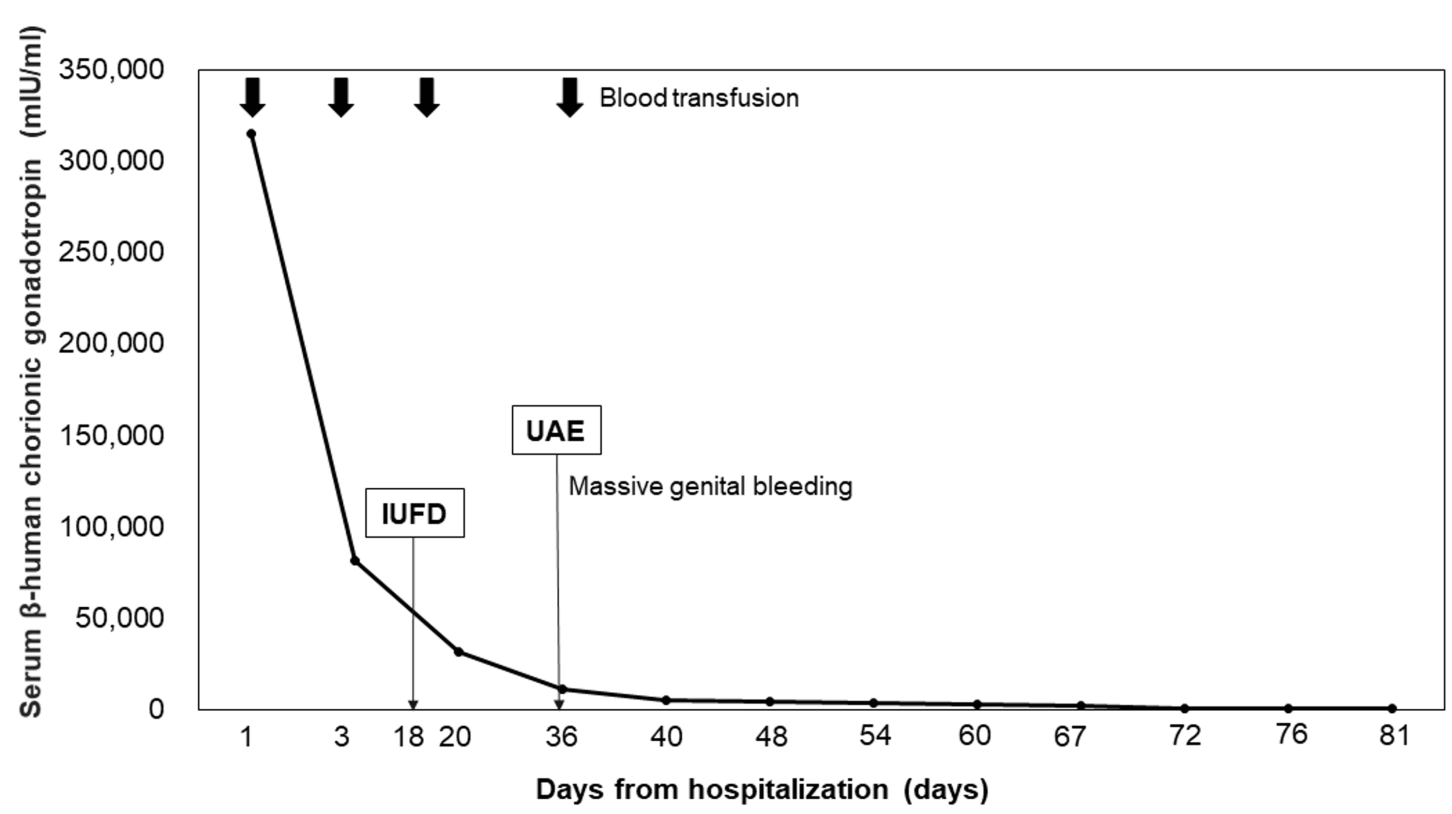
Changes in serum human chorionic gonadotropin during follow-up UAE: uterine artery embolization; IUFD: intrauterine fetal death

We initially anticipated spontaneous evacuation based on our therapeutic strategy. However, with a steady decline in hCG levels (Figure 3), we shifted our approach to expect spontaneous shrinkage. Contrast-enhanced MRI confirmed a further reduction in the fetal and placental components (Figure 2B), and the patient was discharged on day 82. Four months later, a follow-up MRI showed no abnormal blood flow in the area previously suspected of having placenta percreta (Figure 2C), and her serum hCG level was 942 mIU/mL.

Ten months later, contrast-enhanced MRI revealed that the villous tissue had nearly disappeared, the uterus had returned to its normal size (Figure 2D), and serum hCG levels were not detectable. Twenty months later, a follow-up contrast-enhanced MRI showed an area of decreased contrast in the uterine fundus and a thinned portion of the myometrium, consistent with the location where the placental villi had previously perforated the myometrium (Figure 2E). Although the placenta percreta almost completely resolved spontaneously, the muscular layer of the perforated area may have sustained damage, resulting in an anatomical defect.

After a detailed explanation of the situation, an exploratory laparoscopy was scheduled. During laparoscopy, a fissure was identified in the uterine fundus near the right uterine horn. The fissure was repaired using two layers of sutures. Four months after laparoscopy, a contrast-enhanced MRI revealed that the myometrium had healed, resulting in a normal-sized uterus with a fully intact myometrial layer around the entire circumference (Figure 2F), allowing pregnancy and planning for embryo transfer.

## Discussion

A woman with SLE and a 20-year history of long-term steroid use became pregnant after vitrified-warmed embryo transfer. At 10 weeks of gestation, she experienced intra-abdominal bleeding due to a suspected placenta percreta perforation. Despite the difficulty in diagnosing uterine perforation at an early stage, the fetus and placenta spontaneously absorb over two years. The uterus subsequently returned to its normal size, and the rupture site was identified and repaired laparoscopically. This case highlights the successful conservative management of placenta percreta, preserving fertility through natural observations.

Recent studies have identified assisted reproductive technology (ART) pregnancies and cryopreserved embryo transfer as independent risk factors for PAS [5-7]. Our patient displayed none of the traditional risk factors but was impregnated via IVF; specifically, vitrified-warmed embryo transfer under the HRT cycle [3]. In the natural menstrual cycle, the corpus luteum following ovulation produces estrogen and progesterone and also releases vasoactive products such as relaxin [8]. In addition, several studies have demonstrated that relaxin induces the production of the decidualization markers insulin-like growth factor-binding protein 1 and prolactin [9]. However, vitrified-warmed embryo transfer during an HRT cycle has not been observed in the corpus luteum. One of the factors thought to cause PAS in embryo transfers during HRT cycles is the lack of corpus luteum at the time of pregnancy establishment [10-12].

In this case, long-term glucocorticoid use, as in patients with SLE, was associated with an increased risk of PAS. Glucocorticoids can impair protein synthesis [13], leading to muscle degeneration and potential weakening of the uterine wall [14,15]. Moreover, glucocorticoids have been demonstrated to reduce natural killer cell numbers at the implantation site [16]. Glucocorticoids have been reported to affect uterine spiral artery maturation during placentation [17]. In the present case, the patient's long-term use of glucocorticoids combined with ART pregnancy following vitrified-warmed embryo transfer during an HRT cycle may have contributed to the development of placenta percreta.

Diagnosis of PAS in early pregnancy is difficult, and many imaging features of PAS have been reported on ultrasonography and MRI scans. Recently, the features of ultrasound findings associated with PAS have begun to be defined, including myometrial thinning, the existence of placenta previa, disappearance of hypoechoic retroplacental area, hypervascularity of the uterovesical plane, disturbance of bladder wall irregularity, presence of bridging vessels (vessels that appear to extend from the placental bed across the uterine wall into the bladder or other pelvic organs), numerous placental lacunae (resulting in an overall "moth-eaten" appearance of the placenta), and placental bulging (ballooning of the uterus containing the placenta into adjacent pelvic structures) [18]. However, none of these imaging findings were extended to change with each week of gestation. True-positive cases of PAS on ultrasound diagnosis are considered to be more positive after 16 weeks of gestation [19], and there is no consensus on the diagnosis before that time [18].

The diagnosis of PAS using MRI is not recommended. According to the American College of Obstetricians and Gynecologists and the Society of Maternal-Fetal Medicine Obstetric Care Consensus, MRI is not the method of choice for the first evaluation of PAS. Its principal use is in cases of posterior placenta and to assess the depth of invasion in suspected placenta percreta [20]. In the present case, MRI revealed that a part of the placenta bulged out of the uterus, which we believe is useful for the diagnosis. MRI is primarily useful for confirming placental resorption.

Laparoscopic and robot-assisted hysterectomies are used for delayed interval management of PAS after third-trimester delivery with residual placenta [21]. Recently, a successful fetal laparoscopic hysterectomy in situ was performed to manage placenta percreta that caused uterine rupture in the first trimester [22]. In the present case, perforation of the placenta percreta was repaired laparoscopically because the area where the perforation was absorbed left damage in the myometrium. This finding suggests that even in cases where a total hysterectomy, such as a PAS, is the only option, fertility-sparing treatment is possible with long-term observation of around 20 months and laparoscopic surgery.

## Conclusions

We encountered a case of intra-abdominal hemorrhage due to placenta percreta perforation at 10 weeks of gestation in a woman with SLE and infertility who became pregnant via vitrified-warmed embryo transfer during an HRT cycle. Conservative management, including UAE, led to complete resorption of the placental site two years later. Although spontaneous placental perforation in early pregnancy is rare, the risk of PAS increases in ART pregnancies, especially during HRT cycles with vitrified-warmed embryo transfer. Additionally, pregnancies in infertile patients treated with oral steroids are at a high risk of PAS because of potential myometrial weakening. Therefore, pretreatment counseling regarding the risks of ART pregnancy and early monitoring of the implantation site is crucial for patients undergoing ART.

## References

[1] Silver RM, Branch DW (2018). Placenta accreta spectrum. N Engl J Med.

[2] Jang DG, Lee GS, Yoon JH, Lee SJ (2011). Placenta percreta-induced uterine rupture diagnosed by laparoscopy in the first trimester. Int J Med Sci.

[3] Cho MK, Ryu HK, Kim CH (2017). Placenta percreta-induced uterine rupture at 7th week of pregnancy after in vitro fertilization in a primigravida woman: case report. J Emerg Med.

[4] Nardi E, Seravalli V, Abati I, Castiglione F, Di Tommaso M (2023). Antepartum unscarred uterine rupture caused by placenta percreta: a case report and literature review. Pathologica.

[5] Fitzpatrick KE, Sellers S, Spark P, Kurinczuk JJ, Brocklehurst P, Knight M (2012). Incidence and risk factors for placenta accreta/increta/percreta in the UK: a national case-control study. PLoS One.

[6] Esh-Broder E, Ariel I, Abas-Bashir N, Bdolah Y, Celnikier DH (2011). Placenta accreta is associated with IVF pregnancies: a retrospective chart review. BJOG.

[7] Kaser DJ, Melamed A, Bormann CL (2015). Cryopreserved embryo transfer is an independent risk factor for placenta accreta. Fertil Steril.

[8] Conrad KP, Baker VL (2013). Corpus luteal contribution to maternal pregnancy physiology and outcomes in assisted reproductive technologies. Am J Physiol Regul Integr Comp Physiol.

[9] Tang M, Mazella J, Zhu HH, Tseng L (2005). Ligand activated relaxin receptor increases the transcription of IGFBP-1 and prolactin in human decidual and endometrial stromal cells. Mol Hum Reprod.

[10] Fujita T, Yoshizato T, Mitao H (2024). Risk factors for placenta accreta spectrum in pregnancies conceived after frozen-thawed embryo transfer in a hormone replacement cycle. Eur J Obstet Gynecol Reprod Biol.

[11] Matsuzaki S, Nagase Y, Takiuchi T (2021). Antenatal diagnosis of placenta accreta spectrum after in vitro fertilization-embryo transfer: a systematic review and meta-analysis. Sci Rep.

[12] Jwa SC, Tamaru S, Takamura M, Namba A, Kajihara T, Ishihara O, Kamei Y (2024). Assisted reproductive technology-associated risk factors for placenta accreta spectrum after vaginal delivery. Sci Rep.

[13] Rabin DS, Johnson EO, Brandon DD, Liapi C, Chrousos GP (1990). Glucocorticoids inhibit estradiol-mediated uterine growth: possible role of the uterine estradiol receptor. Biol Reprod.

[14] Tomimatsu T, Hazama Y, Takeuchi M, Kimura T, Shimoya K (2018). Unresponsiveness to oxytocin due to an extremely thin uterine wall in a pregnant woman with systemic lupus erythematosus and Sjögren's syndrome. J Obstet Gynaecol.

[15] Mitoma T, Hayata K, Yokohata S (2022). Diffuse myometrium thinning and placenta accreta spectrum in a patient with systemic lupus erythematosus (SLE): a case report and review of the literature. BMC Pregnancy Childbirth.

[16] Quenby S, Kalumbi C, Bates M, Farquharson R, Vince G (2005). Prednisolone reduces preconceptual endometrial natural killer cells in women with recurrent miscarriage. Fertil Steril.

[17] Lash GE, Bulmer JN, Innes BA, Drury JA, Robson SC, Quenby S (2011). Prednisolone treatment reduces endometrial spiral artery development in women with recurrent miscarriage. Angiogenesis.

[18] Jauniaux E, D'Antonio F, Bhide A (2023). Modified Delphi study of ultrasound signs associated with placenta accreta spectrum. Ultrasound Obstet Gynecol.

[19] Bowman ZS, Manuck TA, Eller AG, Simons M, Silver RM (2014). Risk factors for unscheduled delivery in patients with placenta accreta. Am J Obstet Gynecol.

[20] Cahill AG, Beigi R, Heine RP, Silver RM, Wax JR (2018). Placenta accreta spectrum. Am J Obstet Gynecol.

[21] Rupley DM, Tergas AI, Palmerola KL, Burke WM (2016). Robotically assisted delayed total laparoscopic hysterectomy for placenta percreta. Gynecol Oncol Rep.

[22] Lee F, Zahn K, Knittel AK, Morse J, Louie M (2020). Laparoscopic hysterectomy to manage uterine rupture due to placenta percreta in the first trimester: a case report. Case Rep Womens Health.

